# Effects of *Agaricus blazei* Murrill polysaccharides on hyperlipidemic rats by regulation of intestinal microflora

**DOI:** 10.1002/fsn3.1568

**Published:** 2020-04-19

**Authors:** Yuxin Li, Xuechun Lu, Xiao Li, Xiao Guo, Yu Sheng, Yingna Li, Guangyu Xu, Xiao Han, Liping An, Peige Du

**Affiliations:** ^1^ Pharmaceutical Analysis College of Pharmacy Beihua University Jilin China; ^2^ General Hospital of People's Liberation Army Beijing China

**Keywords:** *Agaricus blazei* Murrill, hyperlipidemia, intestinal microflora, polysaccharide

## Abstract

The present research envisaged the effects of *Agaricus blazei* Murrill polysaccharides (ABPs) on blood lipids and its role in regulation of the intestinal microflora in hyperlipidemic rats. The acidic polysaccharide fraction of *Agaricus blazei* Murrill was obtained by DEAE‐cellulose ion exchange column chromatography. The sugar content of ABP was 75.1%. Compared with the model group (MG), the serum TC, TG, and LDL‐C levels decreased (*p* < .05 or *p* < .01) and the HDL‐C levels increased (*p* < .01) significantly in the ABP group. Expression of CYP7A1 was up‐regulated (*p* < .01), and that of SREBP‐1C (*p* < .05) was down‐regulated significantly in the liver tissue of rats in the ABP group. Additionally, the disordered hepatic lobules and the steatosis of hepatocytes were found to be significantly alleviated in the ABP group. We believe that ABP can reduce the ratio of Firmicutes/Bacteroidetes and reduce the relative abundance of Firmicutes, Ruminococcaceae_unclassified, and Ruminococcaceae, increasing the relative abundance of Proteobacteria, Clostridium_sensu_stricto, Allobaculum, Peptostreptococcaceae, Clostridiaceae_1, and Erysipelotrichaceae as targets to regulate blood lipids. The results showed ABP could regulate the dyslipidemia in rats with hyperlipidemia. The mechanism may be through the regulation of the imbalance of intestinal microflora induced by the high‐fat diet in rats, which may be one of the important ways of its intervention on the dyslipidemia induced by high‐fat diet.

## Graphical abstract

Agaricus blazei Murrill polysaccharides (ABPs) could regulate the blood lipids in hyperlipidemic rats. The mechanism may be through the regulation of the imbalance of intestinal microflora induced by the high‐fat diet in rats, which may be one of the important ways of its intervention on the dyslipidemia induced by high‐fat diet.

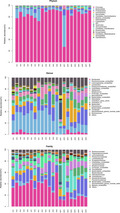

## 1 INTRODUCTION

Hyperlipidemia has become a global concern, often associated with a variety of chronic diseases. Although the genetic and environmental factors have made individuals more likely to gain weight, the underlying cause of hyperlipidemia is the imbalance between dietary intake and energy consumption (Go & Mani, 2012; Lee et al., 2015). Therefore, more and more attention has been paid to the effective treatment methodologies, especially using the natural products as potential functional antihyperlipidemic components (Kwak, Kyung, Kim, Cho, & Rhee, 2010).

Polysaccharides are biological macromolecules that can offer a good biocompatibility and exhibit less toxic and side effects, thereby attracting a good research attention. A large number of studies have shown that the natural polysaccharides presented many pharmacological activities such as antitumor, hypolipidemic, hypoglycemic, antioxidant, and immune activities, with broad application prospects (Yang et al., 2018; Yu et al., 2013; Zhao, Qian, Yin, & Zhou, 2014). *Agaricus blazei* Murrill (ABM) used in this study is a kind of medicinal and edible fungus, rich in a variety of nutrients and chemicals, such as polysaccharides, phytosterols, saponins, glycoproteins, beta‐D‐glucan, and other phytochemicals (Niwa, Tajiri, & Higashino, 2011). *Agaricus blazei* Murrill polysaccharides (ABPs), one of the main active substances in *Agaricus blazei* Murrill, is known to have antihyperlipidemic, antioxidant, antiradiation damage, immune, and anti‐inflammatory activities (Da Silva et al., 2013). Although it has been reported in the literature that it has a role in regulating blood lipids (Wei et al., 2019), its mechanism is still unclear.

Intestinal microflora is closely related to the occurrence of hyperlipidemia. Patients with hyperlipidemia are often accompanied by the imbalance in the intestinal microflora, which aggravates the body's lipid metabolism disorder and is a vicious cycle (Shuang, Wenfei, & Haitao, 2013). In recent years, a large number of studies have shown that polysaccharides can shape the intestinal microflora to promote the growth and proliferation of the intestinal hyperlipidemia‐related beneficial bacteria and inhibit those of harmful bacteria, thereby regulating and maintaining their normal physiological activities (Kaoutari, Armougom, Gordon, Raoult, & Henrissat, 2013). The products produced by intestinal microflora by degrading polysaccharides such as acetic acid, propionic acid, butyric acid, and lactic acid provide energy for the body and regulate the intestinal pH and microbial diversity, thereby playing an important role in protecting intestinal peristalsis and intestinal barrier (Jang, Ridgeway, & Kim, 2013; Okeke, Roland, & Mullin, 2014). Whether ABP can play a hypolipidemic role through the regulation of intestinal flora needs further research.

In this study, a rat model for hyperlipidemia was established by giving a high‐fat diet to rats for exploring the improvement of ABP on the hyperlipidemia in rats, and the relationship between the hypolipidemic effects of ABP from the perspective of intestinal microflora regulation was studied. The mechanism of the hypolipidemic effect of ABP was revealed, which may provide an experimental basis and theoretical basis for the development of highly processed products of *Agaricus blazei* Murrill.

## 2 MATERIALS AND METHODS

### 2.1 Materials

SPF‐grade male *SD* rats, aged from 4 to 5 weeks and weighing 190.0 ± 2.0 g, were purchased from Changchun Yisi Laboratory Animal Technology Co., Ltd. (license number was SCXK (Ji)‐2016‐0003). A high‐fat diet was obtained from the Jilin Medical College, and the formula is shown in detail in Table 1.

**TABLE 1:** High‐fat diet formula

Ingredients	Mass (kg)
Sucrose	20%
Lard	15%
Cholesterol	2%
Sodium cholate	0.2%
Casein	10%
Calcium hydrogen phosphate	0.6%
Basic feed	52.2%

During the experiments, the rats were fed with a free access to water and food, and acclimated to the environment in the laboratory for 7 days. The temperature of the animal room was 20.0°C–22.0°C, and the relative humidity was 50%–60%.

ABM was purchased from Shenyang Juxin Cordyceps Militaris Industry Co., Ltd; glucose (Glc), fucose (Fuc), galactose (Gal), mannose (Man), and glucuronic acid (GlcA) were from Sigma Company; total cholesterol kit (TC), triglyceride kit (TG), and high‐ and low‐density lipoprotein cholesterol (HDL‐C and LDL‐C) kits were from Nanjing Jiancheng Biotechnology Co., Ltd; RIPA lysis buffer was from Beijing Dingguo Changsheng Biotechnology Co., Ltd; Rat CYP7A1, SREBP‐1C, and GAPDH monoclonal antibodies were from AFFINITY; horseradish peroxidase‐labeled goat anti‐rabbit IgG was from Abcam; Phusion Hot Start flex 2X Master Mix was from Shanghai Yitao Biological Instruments Co., Ltd; DL2000 DNA Marker was from Takara Company; Genecolour was from Beijing Jinboyi Biotechnology Co., Ltd; Qubit dsDNA HS Assay Kit was from Invitrogen, Life Technologies; Biowest Agarose G‐10 was from BIOWEST; 50 × TAE Buffer was from Sangon Biotech; AxyPrep PCR Cleanup Kit was from AXYGEN, Life Science Research; and Stool DNA Kit (200) was from OMEGA Bio‐tek. All other reagents used were of AR grade.

Shimadzu HPLC liquid chromatograph (SHIMADZU International Trade Co., Ltd.); UV‐2550 ultraviolet‐visible spectrophotometer (SHIMADZU International Trade Co., Ltd.); Infinite M200 enzyme labeling instrument (TECAN); electrophoresis apparatus (Bio‐Rad company); full‐automatic fluorescence and chemiluminescence gel imager (Beijing Saizhi Pioneering Technology Co., Ltd.); A200 gene amplifying instrument (Hangzhou Langji Scientific Instrument Co., Ltd.); R EPS3 00 electrophoresis apparatus (Tanon Science and Technology Co., Ltd.); and gel imager (Tanon 2500, Tanon Science and Technology Co., Ltd.) were used for analytical purposes.

### 2.2 Preparation of ABP

The fruiting bodies of ABM were crushed into pieces at room temperature, and the pieces of the fruiting bodies of ABM were prepared to obtain ABP. The fruiting bodies were sieved through 60‐mesh sieve, and the impurities were removed manually. Anhydrous ethanol was added to the powdered ABM and filtered to separate the residue, which was dried to constant weight and extracted with water. The filtrate was concentrated and subjected to alcohol precipitation using anhydrous ethanol for removal of proteins by the Sevage method (left at room temperature overnight). Subsequently, dialysis was performed for the sample with a dialysis bag (retention molecular weight was 3500D and dialyzed in distilled water for 48 hr to remove the small molecular substances). The product was freeze‐dried to obtain crude ABPs, which was fractionated by chromatography on a DEAE‐cellulose ion exchange column to obtain the pure ABP. The analysis of monosaccharide composition of the ABP was done by Shimadzu HPLC (Wu et al., 2014).

### 2.3 Establishment of the hyperlipidemia rat model

Thirty‐two SPF‐grade male *SD* rats were randomly divided into four groups: normal control group (NG), model group (MG), positive drug group (PD), and ABP group (ABP). Rats in the NG group were fed with the general diet, and those in MG, PD, and ABP groups were fed with the high‐fat diet. Rats in the PD group were daily given 8.4 mg/kg lovastatin intragastrically once, those in ABP group were given 640 mg/kg ABP, and those in the NG and MG groups were given an equal volume of distilled water in the same way. The high‐fat feeding and the administration lasted 8 weeks continuously.

### 2.4 Calculations of body weight and organ index

The rats were weighed once a week during feeding, and 8 weeks later, the spleen and liver of rats were taken by dissection and washed with saline, and the wet weights of the spleen and liver were recorded for the calculation of the organ indexes as shown in Equation 1 (Khlifi et al., 2019).

(1) $$\text{Visceral index}\;\left(\%\right)=\frac{\text{visceral mass (mg)}}{\text{animal body weight (g)}}\times 100\%$$

### 2.5 Determination of biochemical indicators

Serum preparation: After the high‐fat feeding and administration for 8 weeks, the rats fasted for 12 hr with a free access to water, and then, their blood samples were collected through the abdominal aorta. The blood samples were centrifuged at 3,500 r/min for 15 min to separate the serum, and the serum samples were kept at −20°C for use. TC, TG, LDL‐C, and HDL‐C contents in the serum of rats were determined according to the manufacturer's instructions.

### 2.6 Detection of Protein Expression by Western Blotting

One hundred milligrams of the liver tissue of each rat in all groups was added with RIPA protein lysis buffer. The liver lysis buffer solution was homogenized, and the homogenate was cracked on ice for 1 hr and then centrifuged at 12,000 r/min for 10 min at 4°C to take the supernatant. The standard curve was established using the BCA method, and the protein concentration in the liver tissue of rats in each group was measured and adjusted. The protein was separated by SDS‐PAGE, in which the loading amount of each sample was 10 µl, and then transferred to a PVDF membrane. 5% skimmed milk powder blocking buffer was added onto the membrane, which was shaken at room temperature for 2 hr. After washing the membrane, the primary antibody dilution solution (CYP7A1: 1:1,000 dilution; SREBP‐1C: 1:1,000 dilution; and NADPH: 1:20,000 dilution) was added onto the membrane, and the membrane was incubated at 4°C overnight. After washing the membrane, the corresponding second rabbit antibody (1:2,000) was added onto the membrane, which was incubated at room temperature for 1 hr. The membrane was washed again and was incubated in ECL luminescent liquid for the development of color; subsequently, the image was photographed and analyzed by an automatic analysis system of electrophoresis gel imaging.

### 2.7 Hematoxylin–eosin (HE) staining

The liver tissue of rats was fixed in 10% formalin solution and then routinely sectioned for the preparation of slices. The slices were stained with HE, and the pathological features of the liver tissue were observed under a light microscope.

### 2.8 Determination of intestinal contents by high‐throughput sequencing

#### 2.8.1 Extraction of total DNA from fecal bacteria

The fecal samples in the rectum of rats (NG, MG, and ABP groups) were collected under aseptic condition and frozen rapidly in liquid nitrogen. The samples were stored at −80°C for use.

The DNA from different samples was extracted using the E.Z.N.A.® Stool DNA Kit (D4015, Omega, Inc.) according to the manufacturer's instructions. The reagent that was designed to uncover DNA from trace amounts of sample was shown to be effective for the preparation of DNA of most bacteria. Nuclear‐free water was used for blank. The total DNA was eluted in 50 µl of elution buffer and stored at −80°C until measurement in the PCR (LC‐Bio Technology Co., Ltd).

### 2.9 PCR amplification and 16S rDNA sequencing

The V3‐V4 region of the prokaryotic (bacterial and archaeal) small‐subunit (16S) rRNA gene was amplified with slightly modified versions of primers 338F (5′‐ACTCCTACGGGAGGCAGCAG‐3′) and 806R (5′‐GGACTACHVGGGTWTCTAAT‐3′) (Fadrosh et al., 2014). The 5′ ends of the primers were tagged with specific barcodes per sample and universal sequencing primers. PCR amplification was performed in a total volume of 25 µl reaction mixture containing 25 ng of template DNA, 12.5 µl PCR Premix, 2.5 µl of each primer, and PCR‐grade water to adjust the volume. The PCR conditions to amplify the prokaryotic 16S fragments consisted of an initial denaturation at 98°C for 30 s; 35 cycles of denaturation at 98°C for 10 s, annealing at 54/52°C for 30 s, and extension at 72°C for 45 s; and a final extension at 72°C for 10 min. The PCR products were confirmed with 2% agarose gel electrophoresis. Throughout the DNA extraction process, ultrapure water was used instead of a sample solution to exclude the possibility of false‐positive PCR results as a negative control. The PCR products were purified by AMPure XP beads (Beckman Coulter Genomics) and quantified by Qubit (Invitrogen). The amplicon pools were prepared for sequencing, and the size and quantity of the amplicon library were assessed on Agilent 2100 Bioanalyzer (Agilent) and with the Library Quantification Kit for Illumina (Kapa Biosciences), respectively. PhiX control library (v3) (Illumina) was combined with the amplicon library (expected at 30%). The libraries were sequenced either on 300PE MiSeq runs, and one library was sequenced with both protocols using the standard Illumina sequencing primers, eliminating the need for a third (or fourth) index read.

### 2.10 Data analysis

Samples were sequenced on an Illumina MiSeq platform according to the manufacturer's recommendations, provided by LC‐Bio. Paired‐end reads were assigned to samples based on their unique barcode and truncated by cutting off the barcode and primer sequence. Paired‐end reads were merged using FLASH. Quality filtering of the raw tags was performed under specific filtering conditions to obtain the high‐quality clean tags according to the fqtrim (V 0.9.7). Chimeric sequences were filtered using Vsearch software (v2.3.4). Sequences with ≥97% similarity were assigned to the same operational taxonomic units (OTUs) by Vsearch (v2.3.4) (Uebanso et al., 2017). Representative sequences were chosen for each OTU, and taxonomic data were then assigned to each representative sequence using the RDP (Ribosomal Database Project) classifier. The differences in the dominant species in different groups and multiple sequence alignment were conducted using the Mafft software (V 7.310) to study phylogenetic relationship of different OTUs. OTU abundance information was normalized using a standard of sequence number corresponding to the sample with the least sequences. Alpha diversity was applied to analyze complexity of species diversity for a sample through 4 indices, including Chao1, Shannon, Simpson, and Observed species. All these indices in our samples were calculated with QIIME (version 1.8.0). Beta diversity analysis was used to evaluate differences in samples in species complexity. Beta diversity was calculated by principal coordinates analysis (PCoA) and cluster analysis by QIIME software (version 1.8.0) (Liu, Wu, & Wang, 2019; Tao et al., 2019).

### 2.11 Statistical methods

All values were expressed as mean ± standard deviation ( $\bar{x}$ ±S). "*n*" was used to represent the number of samples in each group. SPSS software (version 16.0) was used for the statistical analysis. The product used WinWrap Basic, Copyright 1993–2007 Polar Engineering and Consulting, http://www.winwrap.com. *p* < .05 was considered statistically significant.

## 3 RESULTS

### 3.1 Preparation of ABP

#### 3.1.1 Extraction and content determination of ABP

ABP was obtained by the dialysis by employing a 1000‐Da dialysis bag to remove the ash content, and the yield was 4.7% relative to the raw material, of which the sugar content was 75.1%, as shown in Table 2. The monosaccharide composition was composed of 79.1% Glc and is shown in Table 3.

**TABLE 2:** Composition analysis of ABP

Polysaccharide	Yield (%)	Total sugar (%)	Glucuronic acid (%)	Protein (%)	Ash (%)
ABP	4.7	75.1	1.9	5.6	5.1

**TABLE 3:** Monosaccharide composition of ABP

Polysaccharide	Monosaccharide (mol%)				
	Glc	Gal	Man	Fuc	GlcA
ABP	79.1	12.4	4.5	1.3	2.7

#### 3.1.2 Isolation and purification of ABP

ABP consisted of small amount of GlcA (2.7%), which was fractionated by ion exchange chromatography on a DEAE‐cellulose column. The acid sugar fraction was obtained with an elution containing 0.5 M NaCl solution. The yield was 47.5%, as shown in Table 4, and the monosaccharide composition is shown in Table 5.

**TABLE 4:** Composition analysis of ABP

Polysaccharide	Yield (%)	Total sugar (%)	Glucuronic acid (%)	Protein (%)	Ash (%)
ABP	47.5	74.1	8.2	4.8	5.1

**TABLE 5:** Monosaccharide composition of ABP

Polysaccharide	Monosaccharide (mol%)			
	Glc	Gal	Man	GlcA
ABP	87.2	3.3	3.8	5.7

### 3.2 Effects of ABP on the body weight and organ index in rats

**FIGURE 1:**
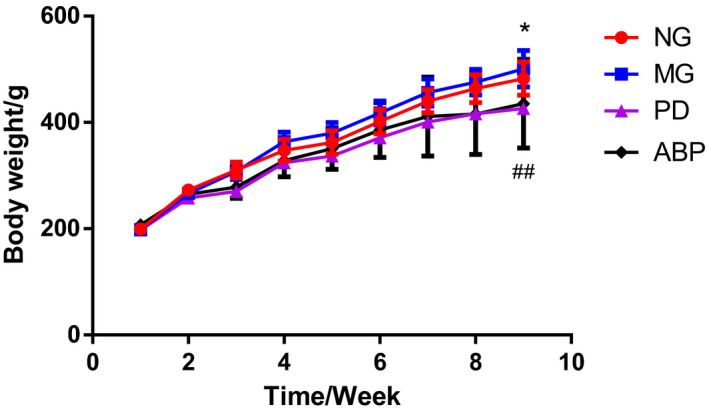
Changes in weight of rats in different groups. *n* = 8; *compared with the NG group, *p* < .05; ^##^compared with the MG group, *p* < .01

There was a persistent increase in the body weight of the rats in the MG group. The increasing trend of body weight of rats in the MG group was almost the same as that in the NG group, but the body weight of rats in the MG group was slightly higher than that in the NG group. On the ninth week, the body weight of rats in the MG group was significantly higher than that in the NG group (*p* < .05). The body weight of rats in the ABP group was significantly lower than that in the MG group (*p* < .01), as shown in Figure 1.

**FIGURE 2:**
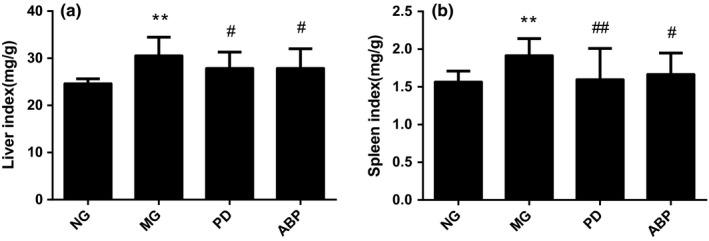
Effect of ABP on organ indexes of hyperlipidemic rats. (a) Effect of ABP on the liver index and (b) effect of ABP on the spleen index. *n* = 8; *compared with the NG group, *p* < .05; **compared with the NG group, *p*< .01; ^#^compared with the MG group, *p* < .05

Compared with those in the NG group, the liver and spleen indexes of rats in the MG group increased significantly (*p* < .01). On the other hand, compared with that of the MG group, the liver index of rats in the PD and ABP groups decreased significantly (*p* < .05). Compared with that of the MG group, the spleen index of rats in the PD group and ABP group decreased significantly with *p* < .01 and *p* < .05, respectively, as shown in Figure 2.

### 3.3 Effect of ABP on biochemical indicators

Compared with the NG group, the levels of TC, TG, and LDL‐C in the serum of rats increased significantly in the MG group (*p* < .05 or *p* < .01), while the levels of HDL‐C decreased significantly (*p* < .05 or *p* < .01). Compared with those in MG group, the levels of TC, TG, and LDL‐C in the serum of rats decreased significantly in the ABP and PD groups (*p* < .05 or *p* < .01), while the HDL‐C levels increased significantly (*p* < .05 or *p* < .01) (Table 6).

**TABLE 6:** Effect of ABP on blood lipid levels in hyperlipidemic rats

Group	TC (mmol/g)	TG (mmol/g)	HDL‐C (mmol/g)	LDL‐C (mmol/g)
NG	1.96 ± 0.14	1.78 ± 0.21	1.84 ± 0.20	1.07 ± 0.04
MG	3.99 ± 0.65^b^	2.76 ± 0.31^a^	1.27 ± 0.13^a^	2.00 ± 0.25^b^
PD	2.10 ± 0.36^c^	1.86 ± 0.16^d^	1.74 ± 0.24^c^	1.33 ± 0.13^d^
ABP	2.17 ± 0.29^d^	2.01 ± 0.25^d^	1.67 ± 0.07^d^	1.36 ± 0.06^d^

Note

*n* = 8.

^a^ Compared with the NG group, *p* < .05.

^b^ Compared with the NG group, *p* < .01.

^c^ Compared with the MG group, *p* < .05.

^d^ Compared with the MG group, *p* < .01.

### 3.4 Effects of ABP on the protein expression of CYP7A1 and SREBP‐1C

**FIGURE 3:**
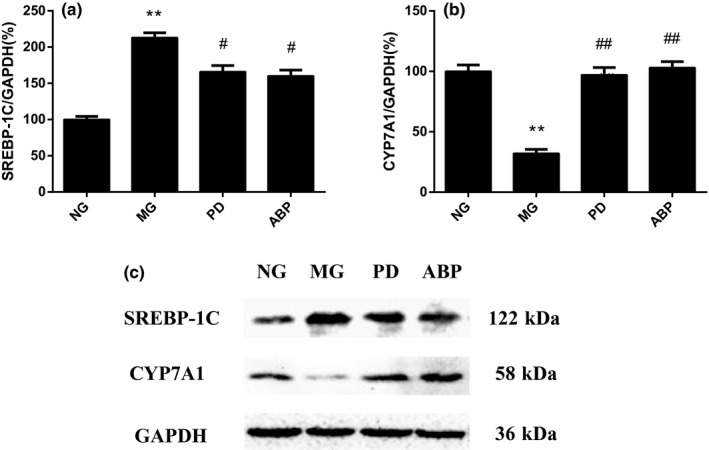
Effects of ABP on the protein expression of CYP7A1 and SREBP‐1C in rat liver tissue. (a) Relative expression of SREBP‐1C protein in the liver of rats; (b) relative expression of CYP7A1 protein in the liver of rats; and (c) electropherogram of hyperlipidemia‐related protein expression in the liver tissue of rats. *n* = 8; *compared with the NG group, *p* < .05; **compared with the NG group, *p* < .01; ^##^compared with the MG group, *p* < .01

Compared with the NG group, the expression of SREBP‐1C protein increased significantly in the MG group (*p* < .01), while the expression of CYP7A1 protein decreased significantly (*p* < .01). The expression of SREBP‐1C protein decreased significantly (*p* < .05 or *p* < .01), and the expression of CYP7A1 protein increased significantly (*p* < .05) in the PD and ABP groups compared with that in the MG group (Figure 3).

### 3.5 Liver HE staining results

**FIGURE 4:**
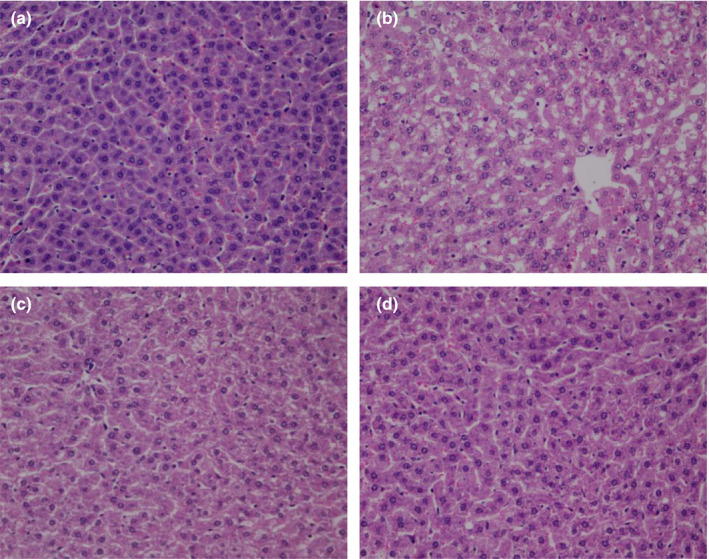
Liver HE staining pathological sections (4 × 100). (a) Liver HE staining results of NG; (b) liver HE staining results of MG; (c) liver HE staining results of PD; and (d) liver HE staining results of ABP

HE staining showed that no abnormal hepatocytes were found, the size of hepatocytes was normal, the hepatic cords were arranged neatly and orderly, and no fat vacuoles and fat infiltration were found in the hepatocytes of rats in the NG group. However, the hepatocytes with lipid‐induced hepatic steatosis were significantly increased, the hepatocytes were extremely swollen and became round, the edema of hepatocytes was noted, the hepatic cords were disordered, and an obvious steatosis of hepatocytes was found in the MG group. The fat vacuoles in the hepatocytes of rats in PD and ABP groups were significantly less than those in the MG group (Figure 4).

### 3.6 Comparative study of intestinal microflora at OTU level in rats

**FIGURE 5:**
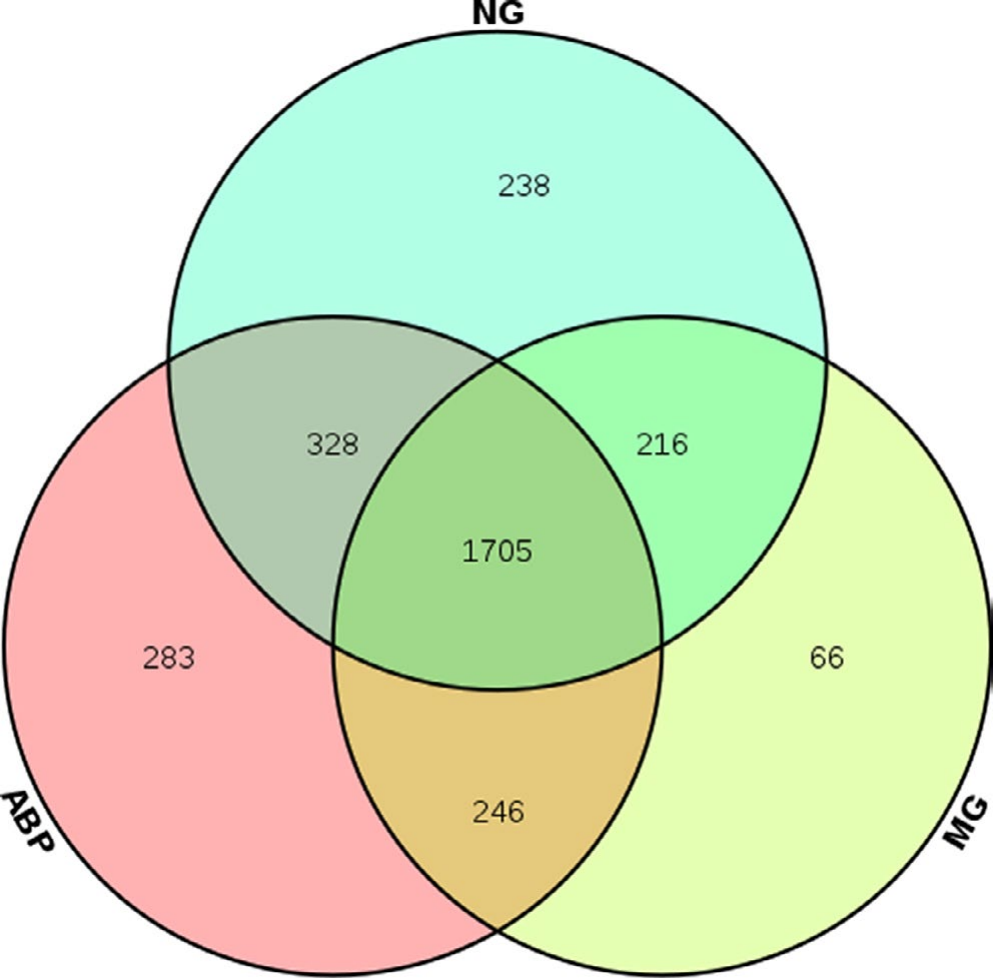
Venn diagram of ABP versus MG versus NG

It was found that 1,705 of 3,507 totally enriched OTUs were shared in all samples. About 48% of the OTUs were not deleted in the ABP group. The order of species richness was as follows: ABP > NG > MG, among which the species richness in the ABP group was highest (Figure 5).

### 3.7 Alpha diversity analysis of intestinal microflora in rats

**FIGURE 6:**
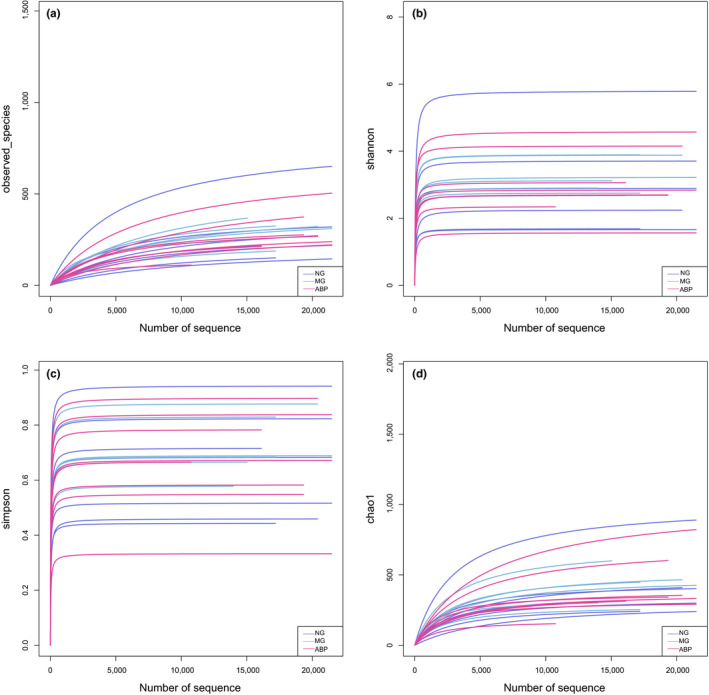
Alpha diversity analysis of intestinal microflora in rats. (a) Observed_species of NG versus MG versus ABP; (b) Shannon of NG versus MG versus ABP; (c) Simpson of NG versus MG versus ABP; and (d) Chao1 of NG versus MG versus ABP

Alpha diversity is usually used to measure the species richness in the community ecology, and it is a comprehensive indicator that reflects the species richness and uniformity. Based on the statistical results of the OTU, the alpha diversity of each sample was calculated, and four diversity indexes were used to analyze the changing trend in the species (Figure 6). The number of OTU species in the sample was sufficient (Figure 6a), the average or uniformity of the abundance of different species in the sample was up to standard (Figure 6b,c), and the amount of sequencing data was saturated (Figure 6d). Observed_species and chao1 indexes showed that NG > ABP > MG. Both the Shannon and Simpson indexes showed that the abundance and uniformity of the ABP group were higher than those of the MG group, and the diversity of the total bacterial community in the NG group was higher (Table 7).

**TABLE 7:** Statistics of the alpha diversity index

Groups	Observed_species	Shannon	Simpson	chao1
NG	1,158.43 ± 65.58	7.77 ± 0.31	0.98 ± 0.01	1,627.46 ± 92.24
MG	902.33 ± 47.98^a^	6.31 ± 0.52^a^	0.93 ± 0.04^a^	1,336.52 ± 120.47^a^
ABP	989.43 ± 181.42^b^	6.89 ± 0.65^b^	0.96 ± 0.02^b^	1,444.28 ± 253.81

Note

*n* = 8.

^a^ Compared with the NG group, *p* < .01.

^b^ Compared with the MG group, *p* < .05.

### 3.8 Beta diversity analysis of intestinal microflora in rats

**FIGURE 7:**
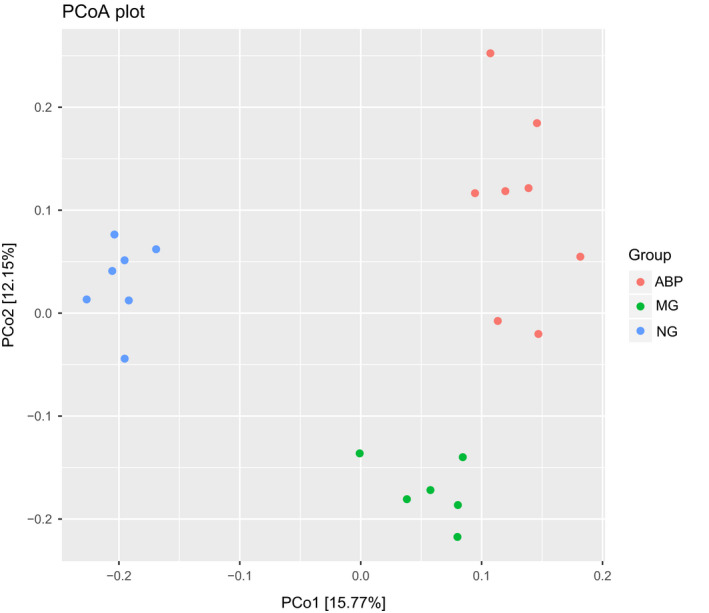
PCoA of ABP versus MG versus NG

In Figure 7, the abscissa represents the first principal component, and the contribution value of the first principal component to the sample difference was found to be 15.77%; the ordinate represents the second principal component, and the contribution value of the second principal component to the sample difference was found to be 12.15%. Each point in the figure represents a sample. The NG group, MG group, and the ABP group can be seen as three independent sets, in which the correlation between the MG group and the ABP group was closer (Figure 7).

### 3.9 Biotaxonomic comparison of intestinal microflora of rats in each group

#### 3.9.1 Comparison at the level of phylum

**FIGURE 8:**
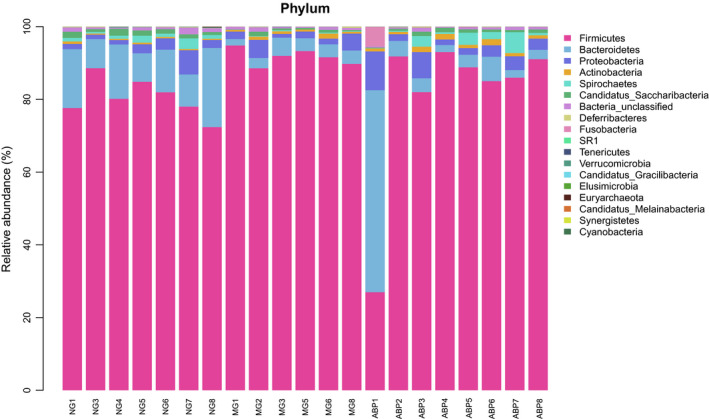
Phylum level of community barplot analysis

Nineteen bacterial floras were identified at the level of phylum in the feces of rats in NG, MG, and ABP groups (Figure 8), including Firmicutes, Bacteroidetes, Proteobacteria, Actinobacteria, Spirochaetes, Candidatus_Saccharibacteria, Bacteria‐unclassified, Deferribacteres, Fusobacteria, SR1, Tenericutes, Verrucomicrobia, Candidatus_Gracilibacteria, Elusimicrobia, Euryarchaeota, Candidatus_Melainabacteria, Synergistetes, and Cyanobacteria.

The statistical analysis at the phylum level on the proportion of the number of phylum sequences in each group accounting to that of the total sequences in the group showed that the relative richness of Firmicutes and Bacteroidetes in the NG, MG, and ABP groups was higher, in which Firmicutes accounted for 80.50%, 91.66%, and 80.58%, respectively, and Bacteroidetes accounted for 12.75%, 3.38%, and 10.04%, respectively. After the treatment with ABP, the relative richness of Firmicutes decreased from 91.66 to 80.58 (*p* < .05), and the ratio of Firmicutes/Bacteroidetes decreased from 27.12 to 8.03 (Table 8).

**TABLE 8:** Top 20 flora and relative abundance ratio at phylum level

	Flora name	NG (%)	MG (%)	ABP (%)
1	Firmicutes	80.50	91.66	80.58
2	Bacteroidetes	12.75	3.38	10.04
3	Proteobacteria	2.62	2.70	4.15
4	Actinobacteria	0.41	0.75	1.11
5	Spirochaetes	1.29	0.15	1.92
6	Candidatus_Saccharibacteria	1.29	0.56	0.81
7	Bacteria_unclassified	0.90	0.66	0.58
8	Deferribacteres	0.10	0.07	0.05
9	Fusobacteria	0.00	0.01	0.70
10	SR1	0.00	0.00	0.00
11	Tenericutes	0.00	0.02	0.02
12	Verrucomicrobia	0.09	0.00	0.01
13	Candidatus_Gracilibacteria	0.00	0.00	0.00
14	Elusimicrobia	0.01	0.00	0.01
15	Euryarchaeota	0.04	0.00	0.00
16	Candidatus_Melainabacteria	0.01	0.02	0.02
17	Synergistetes	0.00	0.00	0.00
18	Cyanobacteria	0.00	0.00	0.00
19	unclassified	0.00	0.00	0.00
20	Others	0.00	0.00	0.00

When compared with the NG group, the relative richness of Firmicutes (*p* < .01) and Actinobacteria (*p* < .01) was higher in the MG group, while the relative richness of Euryarchaeota (*p* < .01), Bacteroidetes (*p* < .01), Spirochaetes (*p* < .01), Candidatus_Saccharibacteria (*p* < .05), and Verrucomicrobia (*p* < .05) was lower in the MG group. Compared with the MG group, the relative richness of Spirochaetes (*p* < .01), Actinobacteria (*p* < .05), and Elusimicrobia (*p* < .05) was higher than in the ABP group (Table 9).

**TABLE 9:** Statistics of top 20 meaningful flora in phylum

Group comparison	Level	Flora name	Abundance change	*p* value
MG/NG	Phylum	Firmicutes	Up	.0043
		Actinobacteria	Up	.0152
		*Euryarchaeota*	down	.0016
		*Bacteroidetes*	down	.0027
		*Spirochaetes*	down	.0027
		*Candidatus_Saccharibacteria*	down	.0152
		*Verrucomicrobia*	down	.0187
ABP/MG	Phylum	Proteobacteria	up	.0109
		Firmicutes	down	.0389
		Spirochaetes	up	.0098

#### 3.9.2 Comparison at the level of genus

**FIGURE 9:**
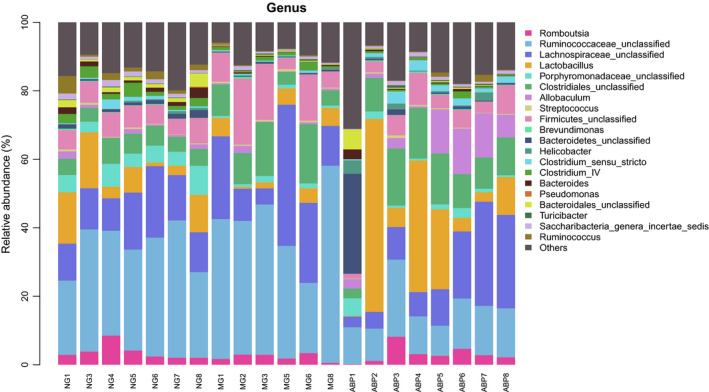
Genus level of community barplot analysis

Twenty bacterial floras were identified at the level of genus in the feces of rats in NG, MG, and ABP groups (Figure 9, Table 10), including Romboutsia, Ruminococcaceae_unclassified, Lachnospiraceae_unclassified, Lactobacillus, Porphyromonadaceae_unclassified, Clostridiales_unclassified, Allobaculum, Streptococcus, Firmicutes_unclassified, Brevundimonas, Bacteroidetes_unclassified, Helicobacter, Clostridium_sensu_stricto, Clostridium_IV, Bacteroides, Pseudomonas, Bacteroidales_unclassified, Turicibacter, Saccharibacteria_genera_incertae_sedis, and Ruminococcus.

**TABLE 10:** Top 20 flora and relative abundance ratio at genus level

	Flora name	NG (%)	MG (%)	ABP (%)
1	Romboutsia	3.70	2.22	3.10
2	Ruminococcaceae_unclassified	31.04	39.13	13.23
3	Lachnospiraceae_unclassified	13.53	19.09	14.08
4	Lactobacillus	8.12	3.66	17.70
5	Porphyromonadaceae_unclassified	5.18	1.06	1.81
6	Clostridiales_unclassified	5.33	9.92	11.15
7	Allobaculum	0.97	0.64	6.65
8	Streptococcus	0.35	0.46	0.33
9	Firmicutes_unclassified	6.12	10.93	5.08
10	Brevundimonas	0.16	0.14	0.17
11	Bacteroidetes_unclassified	1.17	0.37	4.18
12	Helicobacter	0.34	0.13	1.18
13	Clostridium_sensu_stricto	0.90	0.34	1.84
14	Clostridium_IV	2.38	1.05	0.62
15	Bacteroides	1.42	0.22	0.63
16	Pseudomonas	0.05	0.06	0.02
17	Bacteroidales_unclassified	1.59	0.21	0.95
18	Turicibacter	0.15	0.27	0.29
19	Saccharibacteria_genera_incertae_sedis	1.29	0.56	0.81
20	Ruminococcus	1.96	0.24	0.55
21	Others	14.25	9.31	15.64

Compared with the NG group, the relative richness of Porphyromonadaceae_unclassified (*p* < .01), Bacteroidetes_unclassified (*p* < .01), Bacteroides (*p* < .01), Bacteroidales_unclassified (*p* < .01), Ruminococcus (*p* < .01), Saccharibacteria_genera_incertae_sedis (*p* < .05), and Clostridium_IV (*p* < .05) was lower in the MG group. When compared to the MG group, the relative richness of Ruminococcaceae_unclassified (*p* < .01) was lower in the ABP group, and the relative richness of Clostridium_sensu_stricto (*p* < .01) and Allobaculum (*p* < .01) was higher in the ABP group (Table 11).

#### 3.9.3 Comparison at the level of family

**FIGURE 10:**
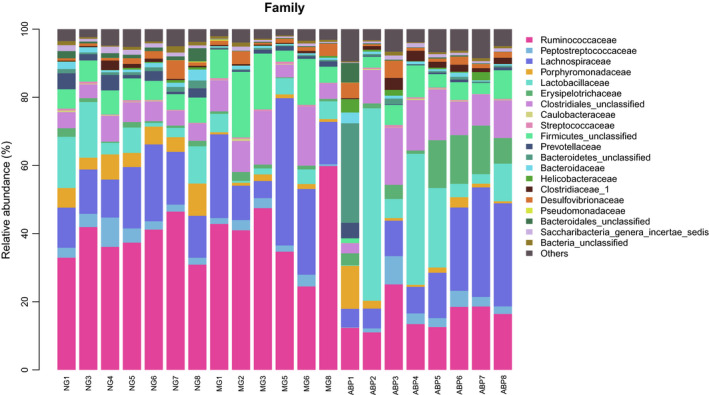
Family level of community barplot analysis

Twenty bacterial floras were identified at the level of family in the feces of rats in NG, MG, and ABP groups (Figure 10), including Ruminococcaceae, Peptostreptococcaceae, Lachnospiraceae, Porphyromonadaceae, Lactobacillaceae, Erysipelotrichaceae, Clostridiales_unclassified, Caulobacteraceae, Streptococcaceae, Firmicutes_unclassified, Prevotellaceae, Bacteroidetes_unclassified, Bacteroidaceae, Helicobacteraceae, Clostridiaceae_1, Desulfovibrionaceae, Pseudomonadaceae, Bacteroidales_unclassified, Saccharibacteria_genera_incertae_sedis, and Bacteria_unclassified. (Table 12).

**TABLE 11:** Statistics of top 20 meaningful flora in genus

Group comparison	Level	Flora name	Abundance change	*p* value
MG/NG	Genus	*Porphyromonadaceae_unclassified*	Down	.0027
		*Bacteroidetes_unclassified*	Down	.0027
		*Bacteroides*	Down	.0027
		*Bacteroidales_unclassified*	Down	.0027
		*Ruminococcus*	Down	.0027
		*Saccharibacteria_genera_incertae_sedis*	Down	.0152
		*Clostridium_IV*	Down	.0223
ABP/MG	Genus	*Ruminococcaceae_unclassified*	Down	.003
		*Clostridium_sensu_stricto*	Up	.0067
		*Allobaculum*	Up	.0098

The statistical analysis at the family level on the number of phylum sequences in each group accounting for that of the total sequences in the group showed that the richness of Ruminococcaceae, Peptostreptococcaceae, and Lachnospiraceae was higher in NG, MG, and ABP groups, in which Ruminococcaceae in the three groups accounted for 38.12%, 41.72%, and 16.00%, respectively; Peptostreptococcaceae 3.72%, 2.24%, and 3.13%, respectively; and Lachnospiraceae 16.21%, 20.09%, and 14.91%, respectively (Table 12).

**TABLE 12:** Top 20 flora and relative abundance ratio at family level

	Flora name	NG (%)	MG (%)	ABP (%)
1	Ruminococcaceae	38.12	41.72	16.00
2	Peptostreptococcaceae	3.72	2.24	3.13
3	Lachnospiraceae	14.91	20.09	16.21
4	Porphyromonadaceae	5.68	1.13	2.81
5	Lactobacillaceae	8.13	3.66	17.72
6	Erysipelotrichaceae	1.21	1.10	7.53
7	Clostridiales_unclassified	5.33	9.92	11.15
8	Caulobacteraceae	0.19	0.16	0.18
9	Streptococcaceae	0.35	0.46	0.33
10	Firmicutes_unclassified	6.12	10.93	5.08
11	Prevotellaceae	2.63	0.93	0.96
12	Bacteroidetes_unclassified	1.17	0.37	4.18
13	Bacteroidaceae	1.53	0.56	1.00
14	Helicobacteraceae	0.34	0.13	1.18
15	Clostridiaceae_1	0.90	0.34	1.84
16	Desulfovibrionaceae	1.80	1.92	2.13
17	Pseudomonadaceae	0.05	0.06	0.02
18	Bacteroidales_unclassified	1.59	0.21	0.95
19	Saccharibacteria_genera_incertae_sedis	1.29	0.56	0.81
20	Bacteria_unclassified	0.90	0.66	0.58
21	Others	4.03	2.86	6.22

Compared with that in the NG group, the relative richness of the Peptostreptococcaceae, Porphyromonadaceae (*p* < .01), Bacteroidetes_unclassified (*p* < .01), Bacteroidales_unclassified (*p* < .01), Bacteroidaceae (*p* < .05), Saccharibacteria_genera_incertae_sedis (*p* < .05), and Prevotellaceae (*p* < .05) was lower in the MG group. Compared with the MG group, the relative richness of Ruminococcaceae (*p* < .01) was lower in the ABP group, while the relative richness of the Peptostreptococcaceae, Clostridiaceae_1 (*p* < .01), and Erysipelotrichaceae (*p* < .01) was higher in the ABP group (Table 13).

**TABLE 13:** Statistics of top 20 meaningful floras in family

Group comparison	Level	Flora name	Abundance change	*p* value
MG/NG	Family	Peptostreptococcaceae	down	.0152
		*Porphyromonadaceae*	down	.0027
		*Bacteroidetes_unclassified*	down	.0027
		*Bacteroidales_unclassified*	down	.0027
		*Bacteroidaceae*	down	.0152
		*Saccharibacteria_genera_incertae_sedis*	down	.0152
		*Prevotellaceae*	down	.0455
ABP/MG	Family	*Ruminococcaceae*	down	.003
		*Peptostreptococcaceae*	up	.0377
		*Clostridiaceae_1*	up	.0067
		*Erysipelotrichaceae*	up	.0098

## 4 DISCUSSION

A hyperlipidemia rat model was established by administering the rats with a high‐fat diet in our laboratory. It was found that the serum TC, TG, and LDL‐C levels of rats increased significantly, the HDL‐C levels decreased significantly, and the liver and spleen indexes increased significantly in the MG group. Serum TC, TG, LDL‐C, and HDL‐C levels are the main indicators to reflect the body's lipid metabolism (Dechesne, Musovic, Palomo, Diwan, & Smets, 2016). Increased TC and LDL‐C are one of the main causes of hyperlipidemia. High‐fat diet can cause the enlargement of liver, while elevated liver and spleen indexes reflect the presence of hyperlipidemia to some extent (Cho et al., 2017). Our results prove that the hyperlipidemia rat model induced by high‐fat diet was successful. After the administration of ABP, the serum TC, TG, and LDL‐C levels of hyperlipidemic rats decreased significantly, while the HDL‐C level increased significantly, and the liver and spleen indexes decreased significantly, suggesting that ABP effectively regulated the blood lipid metabolism in hyperlipidemic rats.

Long‐term high‐fat diet may lead to the dyslipidemia in rats, wherein the levels of TC and TG increase in the blood of the rats. The key enzyme involved in the metabolism of TC is 7α‐hydroxylase (CYP7A1), the rate‐limiting enzyme for the transformation of cholesterol into bile acid in liver, and mainly distributed in liver, small intestine, and adipose tissue (Guo et al., 2011; Hasegawa et al., 2019). It plays an extremely important role in maintaining the dynamic balance of cholesterol and cholic acid (Lee et al., 2018; Lim, Wang, Wessells, Ocorr, & Bodmer, 2011). In this study, the expression of CYP7A1 protein in the MG group decreased, while the expression of CYP7A1 protein increased after the administration of ABP, indicating that ABP could promote the metabolism of cholesterol in the body (Chu et al., 2013). In addition, SREBP‐1C, the key transcription factor of TG metabolism that is generally widely distributed in liver and most other tissues, controls the synthesis of fat and fatty acids, thereby playing an important role in the mechanism of lipid toxicity. The increased expression and activity of SREBP‐1C can cause lipid deposition in the cells and impair the function of liver, skeletal muscle, and adipose tissue to metabolize glucose (Tang et al., 2017). The results showed that the administration of ABP led to an increase in the expression of SREBP‐1C protein and a decrease in the expression of SREBP‐1C in the MG group, which can improve the lipid deposition in the liver tissue, suggesting that ABP could play a hypolipidemic role by activating the key enzyme CYP7A1 in the metabolism of cholesterol and inhibiting the SREBP‐1C factor for the lipid metabolism.

In this study, 16S rDNA high‐throughput sequencing technique was used to analyze the effect of ABP on the intestinal microflora in hyperlipidemic rats. The results of the study on the diversity of bacterial flora showed that there were differences in the number of OTU in rats among the groups. This showed that the bacterial flora between NG, MG, and ABP groups was very different, and a high‐fat diet led to a decrease in the abundance of the fecal flora in rats in the MG group. The abundance of the flora in the ABP group significantly increased along with the flora in the NG group, which can further prove that the ABP changed the number of bacterial OTUs in rats and greatly changed the composition of intestinal flora. Furthermore, the alpha diversity and beta diversity analyses showed that the species richness of the bacterial flora changed in rats of each group, wherein the richness decreased significantly in the MG group while it is increased significantly in the ABP group. This indicates that the high‐fat diet destroyed the balance of the intestinal microflora in rats, while ABP assisted the process of establishment of a relatively stable microflora structure. In this study, Bacteroides and Firmicutes were found to be the dominant flora in all samples. A large reduction in Firmicutes was observed, accompanied by a relative increase in the Bacteroides and Proteobacteria after the administration of ABP to the hyperlipidemic rats, although there were some significant individual differences, and the ratio of Firmicutes/Bacteroidetes decreased accordingly. The ratio of Firmicutes/Bacteroidetes has been considered to reflect the obesity or the ecological imbalance of the gastrointestinal tract and as a representative parameter of health status (Bortolin et al., 2018; Van Driel et al., 2016; Xue et al., 2016). Our results indicated that the Firmicutes may be inhibited by ABP to a large extent, which tends to balance the Bacteroides, suggesting that ABP can improve the expression of the dominant bacteria in the intestinal tract of rats and regulate the composition of the intestinal microflora. The results of this study at the genus classification level indicated that the abundance of the Bacteroides in the MG group was significantly reduced. Wangjiao Tang (Tang et al., 2018) showed that Bacteroides were positively correlated with the TG levels and negatively correlated with the HDL‐C levels, and were the core flora in the cause of hyperlipidemia. Clostridium_IV belongs to the order Clostridium and is known to be a beneficial gut bacterium (Clausen & Mortensen, 1995). It was found that the abundance of Clostridium_IV was significantly reduced, which was consistent with the earlier findings. In the ABP intervention group, the harmful bacteria Ruminococcaceae_unclassified decreased, and there are reports in the literature that Ruminococcaceae is a group of gut flora known to be directly related to obesity (Arumugam et al., 2011; Daniel et al., 2014; Velagapudi et al., 2010). It was observed that the beneficial abundance of Clostridium_sensu_stricto and Allobaculum increased significantly. Clostridium_sensu_stricto belongs to the order Clostridium and can decompose polysaccharides to produce short‐chain fatty acids. It is also known to promote the lipopolysaccharide absorption and inhibit the adipokines induced by intestinal hunger (Clausen & Mortensen, 1995). Allobaculum is a gram‐positive bacterium whose end products are lactic acid and butyric acid, which can improve the nonalcoholic fatty liver (Greetham et al., 2004; Pitts & Van Thiel, 1986). The results of this study suggested that at a more specific family classification level, the abundances of Bacteroidaceae, Porphyromonadaceae, and Prevotellaceae were significantly reduced in the MG group. Bacteroidaceae, Porphyromonadaceae, and Prevotellaceae belong to the order Bacteroides (GARRITY, BELL, & LILBURN, 2004). It has been reported (Guo et al., 2008) that the Bacteroides order has a negative correlation with obese body weight, which is consistent with the results of our studies at the level of phylum and genus. The abundance of harmful bacteria Ruminococcaceae in the ABP intervention group was significantly reduced, which was consistent with the results at the genus level. The abundance of probiotics increased significantly, including that of Peptostreptococcaceae, Clostridiaceae_1, and Erysipelotrichaceae. Peptostreptococcaceae is a gram‐positive and anaerobic coccus, and its main function is to regulate blood lipids (Sookoian et al., 2020). Clostridiaceae_1 belongs to the order Clostridium, which is consistent with the genus‐level study. Chen et al. (2018) found that the polysaccharides of Fuzhuan brick tea can modulate the diversity of small intestinal microorganisms in high‐fat diets, by increasing the relative abundance of Erysipelotrichaceae and reducing the effects of high‐fat diet. It was found that ABP reduced the ratio of Firmicutes/Bacteroidetes, reduced the relative abundance of Firmicutes, Ruminococcaceae_unclassified, and Ruminococcaceae, and increased the relative abundance of Proteobacteria, Clostridium_sensu_stricto, Allobaculum, Peptostreptococcaceae, Clostridiaceae_1, and Erysipelotrichaceae as targets to regulate the blood lipids by regulation of the balance of the intestinal microecological bacteria.

This study clarified the lipid‐lowering activity of ABP and its correlation with the regulation of intestinal flora, thereby laying a foundation for the research on the role and mechanism of the active ingredients of *Agaricus Blazei* Murrill.

## 5 CONCLUSION

ABP has a hypolipidemic effect, which may be related to its ability to regulate the expression of key lipid metabolism‐related factors CYP7A1 and SREPP‐1C. The hypolipidemic effect of *Agaricus blazei* Murrill polysaccharides is related to its modulation and regulation of the imbalance of the intestinal microflora structure.

## CONFLICTS OF INTEREST

The authors declare that they have no conflicts of interest.

## AUTHOR CONTRIBUTION

Yuxin Li conceptualized the idea, executed the experiments, prepared the original draft, and contributed to data curation. Xuechun Lu reviewed and supervised the manuscript. Xiao Li executed experiments. Xiao Guo, Yu Sheng, Yingna Li, Guangyu Xu, and Xiao Han supervised the manuscript. Liping An supervised the manuscript, and contributed to methodology and draft editing. Peige Du supervised the manuscript and contributed to draft editing.

## ETHICAL STATEMENT

The animal experiments were approved by the Institutional Animal Care and Use Committee (IACUC) of Beihua University. All of the experimental procedures were performed in accordance with the Guide for the Care and Use of Laboratory Animals (China).
